# Supplementary material for: Pancreatic cancers suppress negative feedback of glucose transport to reprogram chromatin for metastasis
Source: Nat Commun. 2020 Aug 13;11:4055. doi: 10.1038/s41467-020-17839-5 (PMC7426874; doi:10.1038/s41467-020-17839-5)
Supplement: Supplementary file 1 — Supplementary Information [file 41467_2020_17839_MOESM1_ESM.pdf]

Pancreatic cancers suppress negative feedback of glucose transport to reprogram chromatin for metastasis

Supplementary Information

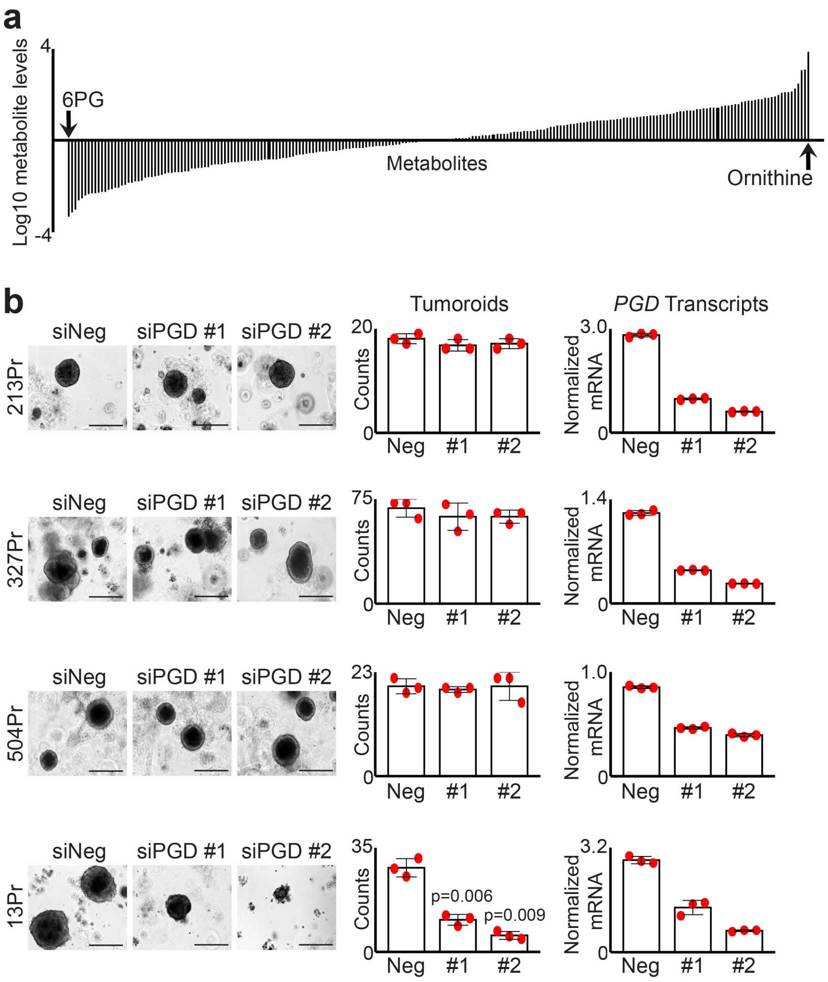

Supplementary Figure 1 6PG depletion in PGD-dependent cell lines and identification of PGD-independent primary tumor samples. a, Plot of $\log_{10}$ transformed metabolite levels (LC-MS) measured in PGD-dependent cell lines (defined by growth response to Crispr/Cas) from the cancer cell line encyclopedia. 6PG was the most depleted metabolite, while ornithine was the most enriched. **b,** RNAi knockdown of PGD (ref.[4,5]) did not impair 3D tumoroid growth of the indicated primary tumor cell lines (top three) with high TXNIP expression (Fig. 1d). In contrast, PGD knockdown strongly impaired tumoroid growth in the control PGD-dependent primary tumor line (13Pr). n=3 technical replicates, error bars: s.d.m., indicated p-values calculated by two-tailed t-tests. Scale bars: 400μm.

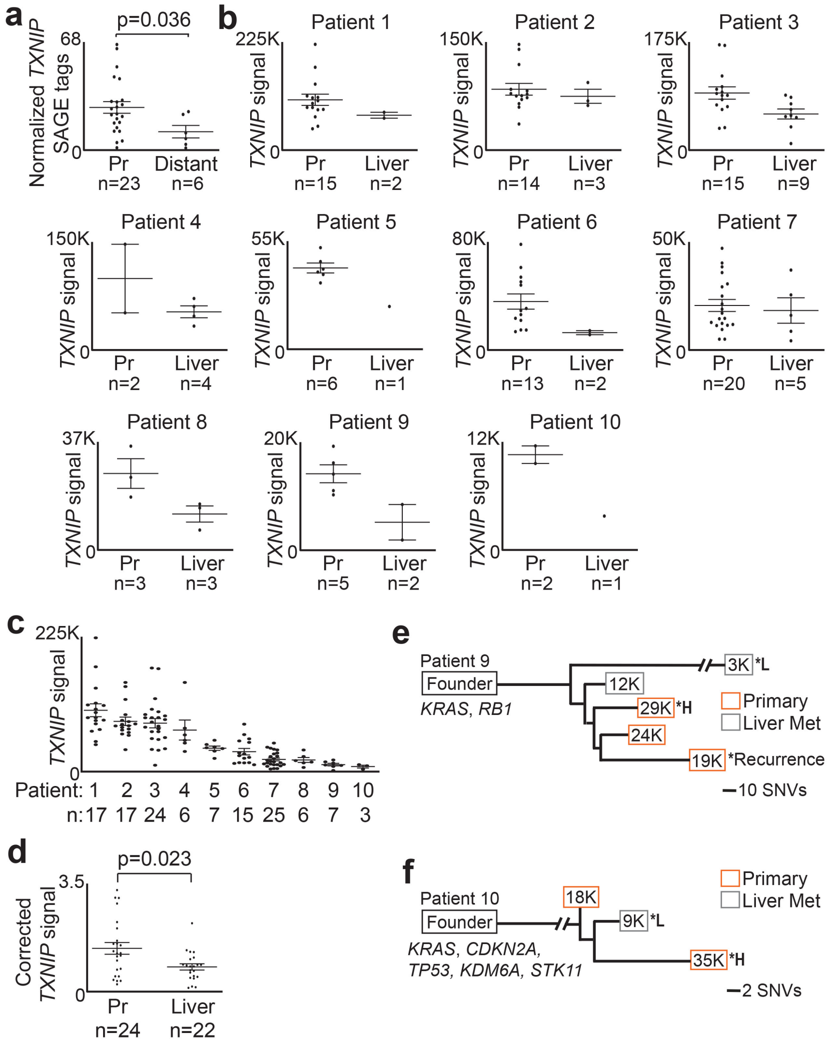

Supplementary Figure 2 ***TXNIP* transcript expression in PDAC patient tissues. a**, *TXNIP* transcript levels are plotted from serial analysis of gene expression (SAGE) tag datasets generated on bulk primary tumor samples (Pr, n=23) and a subset of the rapid autopsy distant metastases (Distant, n=6) (ref.[16]). *TXNIP* tag signals were normalized by dividing values by the total number of tags in each sample. TXNIP expression was significantly lower overall in the metastases relative to the pooled primary tumors (p=0.036 by two-sided Mann-Whitney *U* tests). **b**, *TXNIP* transcript levels detected from matched regions of primary tumors (Pr) and individual liver metastases (Liver) for each of the rapid autopsy patients included in Fig. 1e-f. Data was plotted from previously generated RNA-seq datasets[34] (error bars: s.e.m., n: total numbers of samples for each patient). **c**, TXNIP transcript levels are plotted from all tumor samples (primary + liver) for each patient on the same x-axis, revealing significant differences in baseline expression between patients (p<0.0001 by one-way ANOVA or Kruskal-Wallis tests). **d,** Baseline corrected *TXNIP* transcript levels (from Fig. 1f) were further corrected by dividing the expression values by the estimated tumor cell purity for samples with available estimates (ref.[34]). *TXNIP* was significantly down-regulated in liver metastases (Liver) compared to primary tumors (Pr) (error bars: s.e.m., p=0.023 by two-sided Mann-Whitney *U* tests). **e-f,** *TXNIP* expression values (for samples with available purity estimates) are indicated for two additional patients with available phylogenetic trees from WGS data (ref.[34]), as in Fig. 1g.

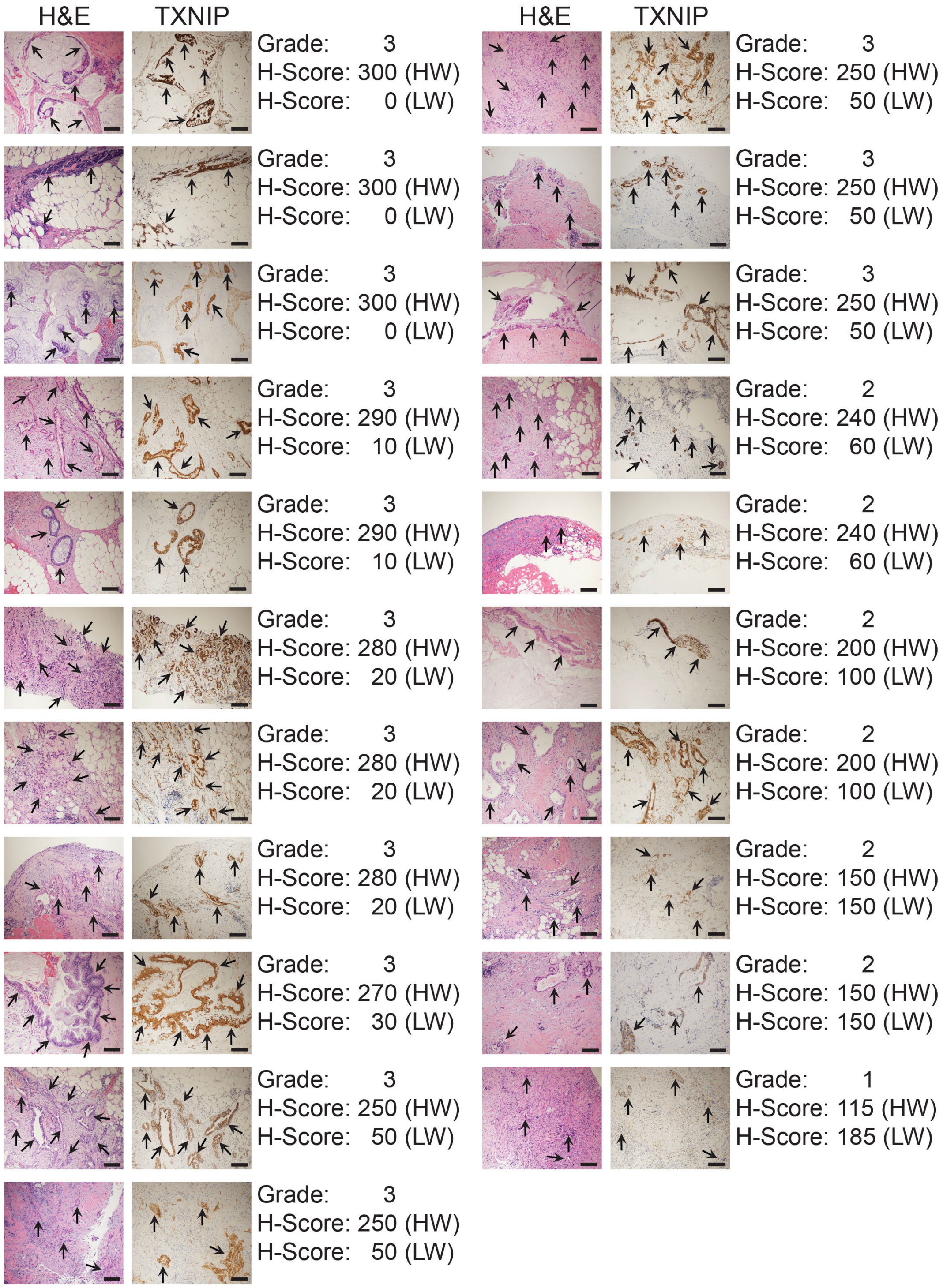

Supplementary Figure 3 TXNIP is expressed in metastatic peritoneal deposits. TXNIP IHC stains (right panels) indicate that most peritoneal metastases uniformly express high to intermediate amounts of TXNIP protein (brown: TXNIP), as reflected in high grades, large high weighted (HW) H-scores, and small low weighted (LW) H-scores. Arrows highlight individual tumor glands embedded within dense stroma or mucin, with corresponding H&E stains (left panels) provided for morphologic comparison (n=21 patient samples, scale bars: 100µm).

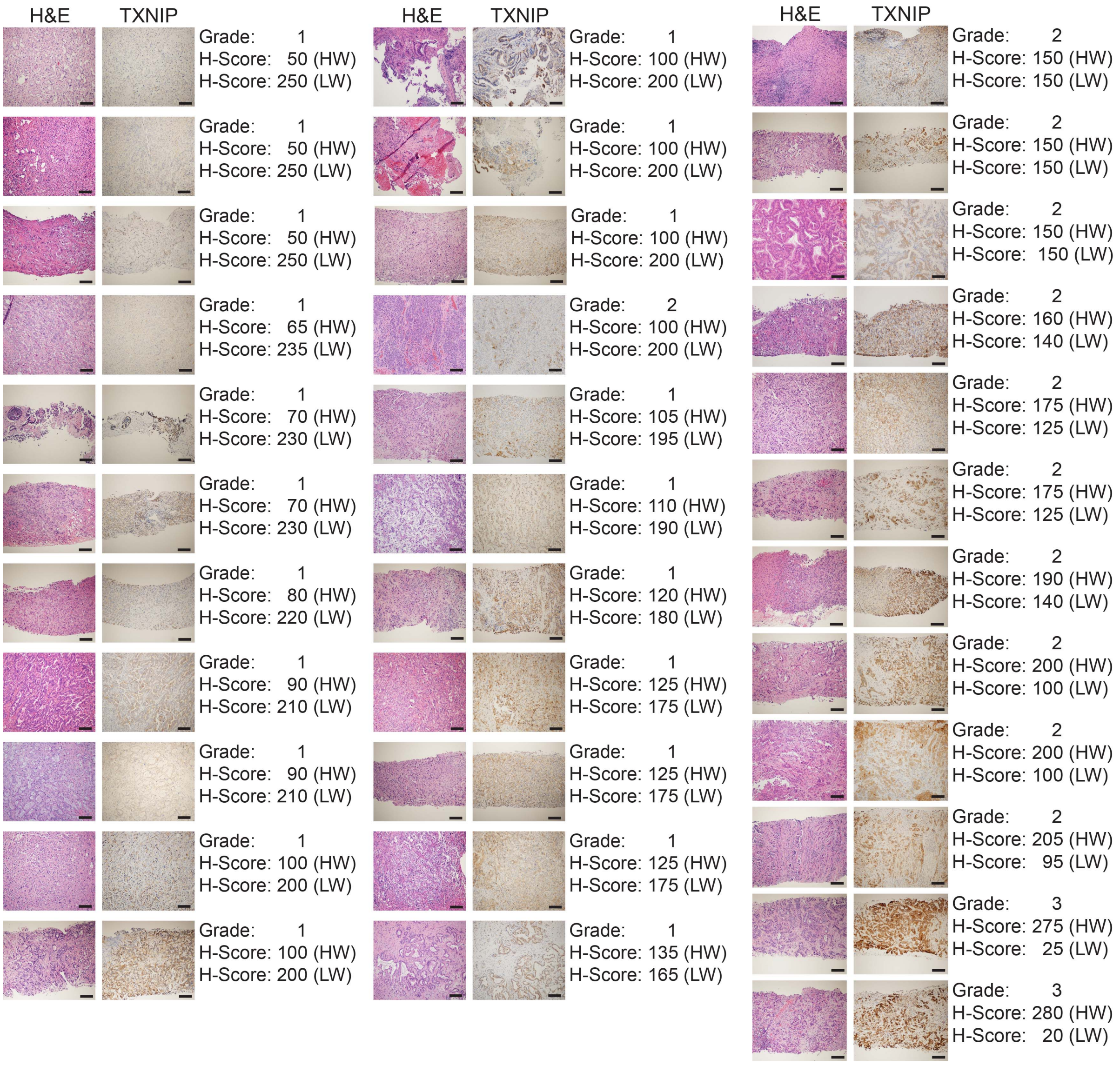

Supplementary Figure 4 TXNIP is suppressed in distant metastases. TXNIP IHC stains (right panels) indicate that most distant metastases uniformly express low to intermediate or heterogenous amounts of TXNIP protein (brown staining), as reflected in low grades, small high weighted (HW) H-scores, and large low weighted (LW) H-scores. Distant metastases do not require arrows to point out tumor as the cellularity is typically much greater than the stroma content, as demonstrated by the corresponding H&E stains (n=34 patient samples, scale bars: 100µm).

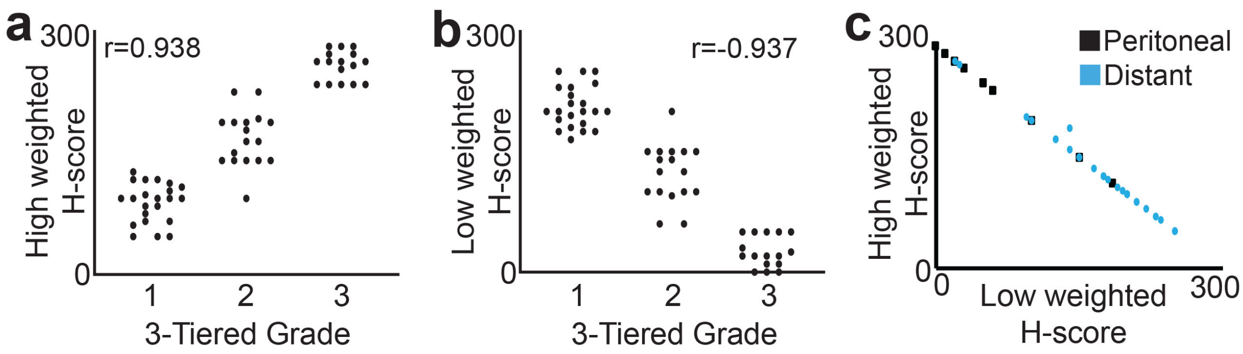

Supplementary Figure 5 Strong agreement between grading and scoring schemes.
a, 3-tiered grades (x-axis) plotted by their corresponding high weighted H-scores (y-axis) for all analyzed samples (n=55, Fig. 1g, Supplementary Fig. 3, 4) shows strong correlation (Pearson's r) between high grade and HW H-score. **b**, 3-tiered grades (x-axis) plotted by their corresponding low weighted H-scores (y-axis) for all analyzed samples (n=55) shows strong anticorrelation (Pearson's r) between high grade and high LW H-score. **c**, High and low weighted H-scores for all samples (n=55) plotted against each other shows that most peritoneal metastases (black) cluster towards the far top left (high expression), whereas most distant metastases (blue) cluster towards the middle to far bottom right (intermediate to low expression).

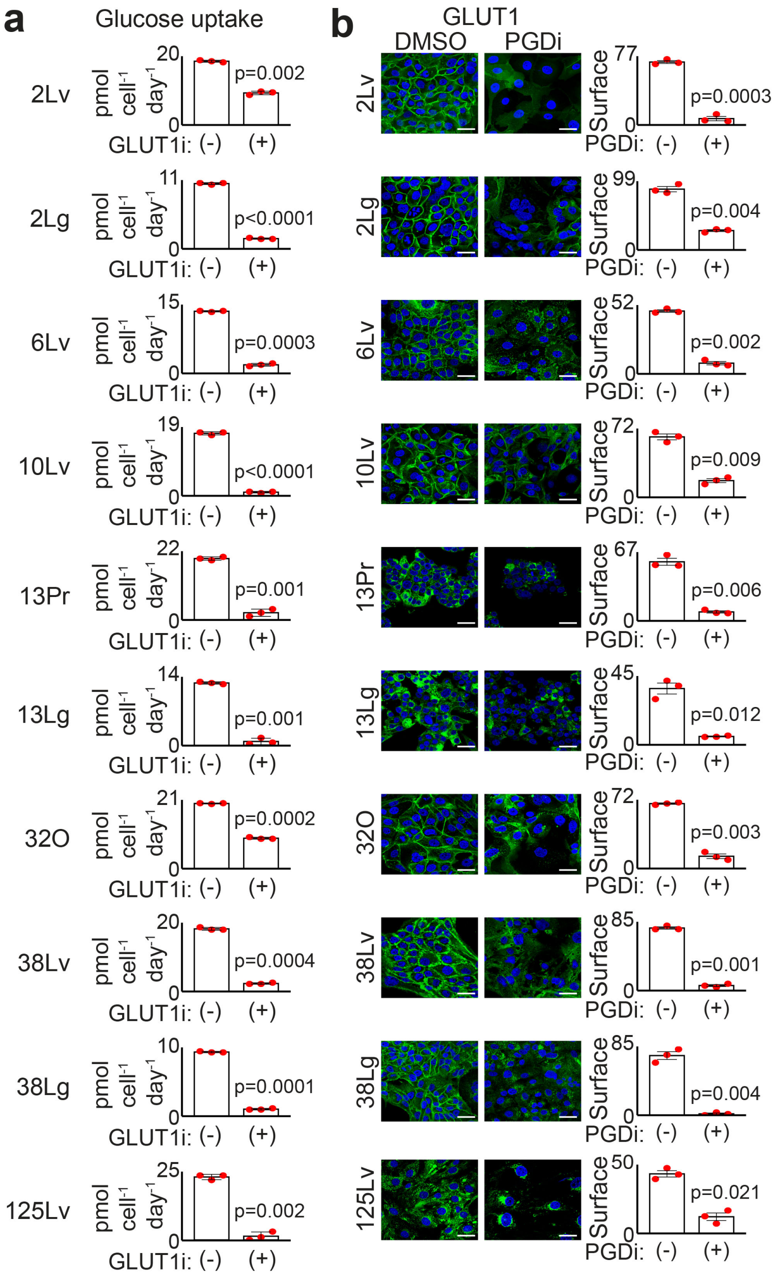

Supplementary Figure 6 Glucose import through surface GLUT1 is PGD-dependent in PGD^{high} PDACs. **a**, GLUT1 inhibitor (GLUT1i) treatments ((-): DMSO, (+): BAY-876) recurrently slowed glucose consumption rates across the indicated PGD^{high} rapid autopsy subclones (n=3 technical replicates; error bars: s.d.m.; indicated p-values calculated by two-tailed t-tests). **b**, PGD inhibitor (PGDi) treatments ((-): DMSO, (+): 6AN) recurrently displaced GLUT1 off the cell surface across the indicated PGD^{high} rapid autopsy subclones (green: GLUT1 IF signals, blue: nuclear Hoescht signals, n=3 biological replicates; error bars: s.e.m.; indicated p-values calculated by two-tailed t-tests, scale bars: 20μm).

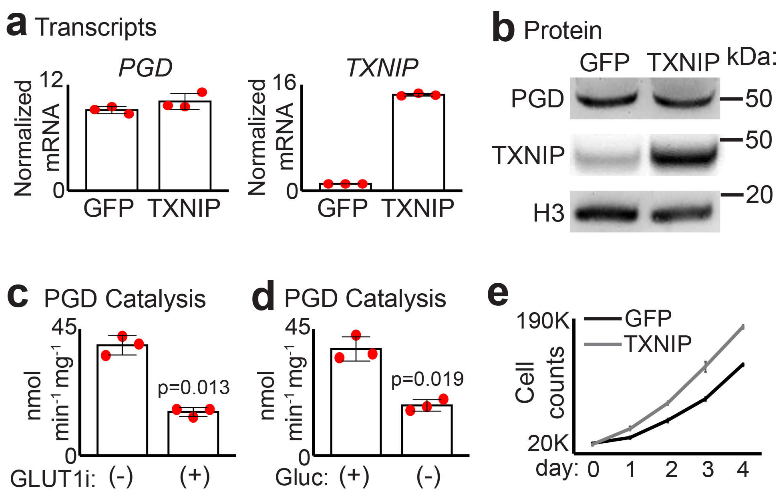

Supplementary Figure 7 High PGD catalysis is dependent on avid glucose uptake. **a**, RT-qPCR measurements indicate that *PGD* mRNA expression (left) is not influenced by exogenous *TXNIP* (right) in PGD^{high} 38Lg cells (n=3 technical replicates, error bars: s.d.m.). **b**, Western blots indicate that PGD protein expression (top) is also insensitive exogenous TXNIP (middle) Blots are representative of two biological replicates. **c**, **d**, Treatment of PGD^{high} cells with either GLUT1 inhibitor (GLUT1i: BAY-876) or glucose starvation (Gluc) significantly slowed PGD catalytic rates (n=3 technical replicates, error bars: s.d.m., p=0.013 and 0.019 by two-tailed t-tests as indicated). **e**, Exogenous TXNIP did not impair 2D growth rates of PGD^{high} cells relative to GFP controls (n=3 technical replicates per time point, error bars: s.d.m.).

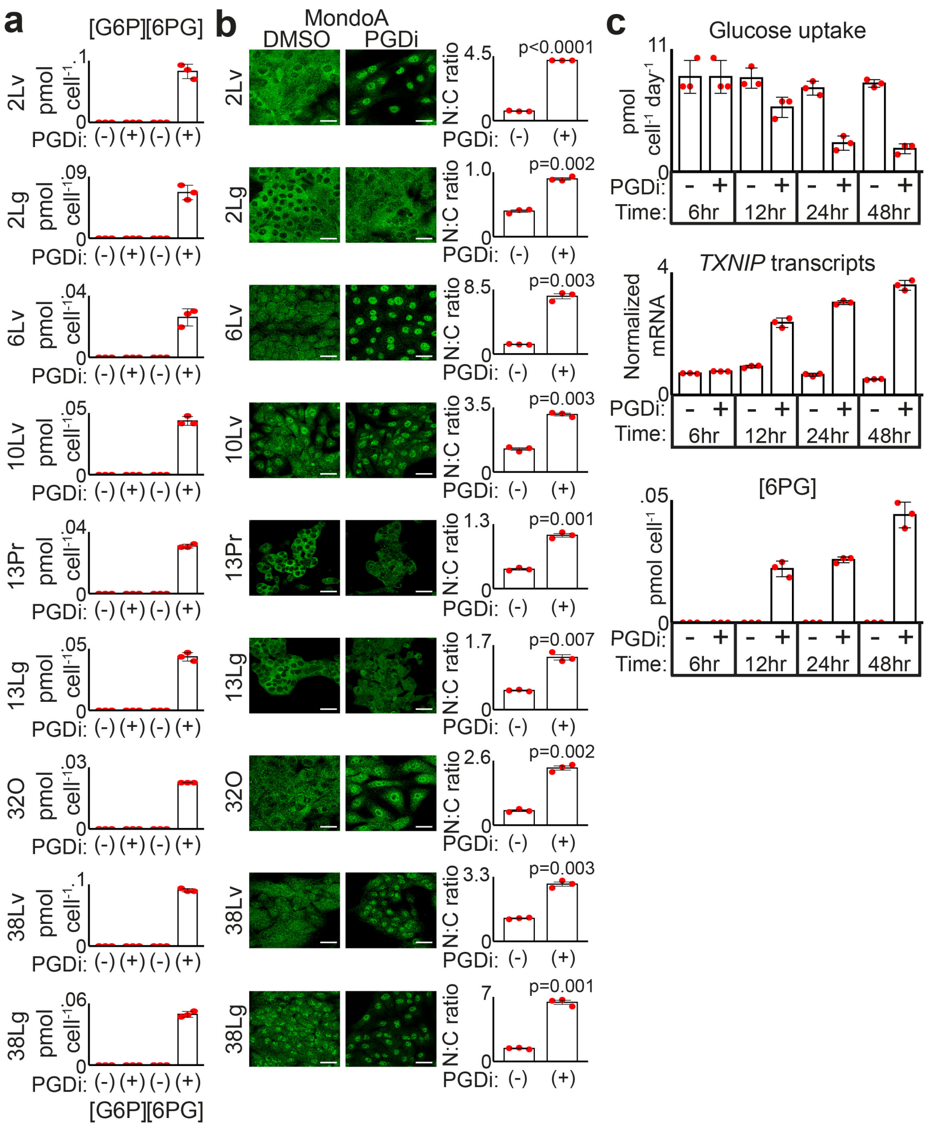

Supplementary Figure 8 PGD inhibition causes 6PG accumulation with nuclear concentration of MondoA. **a**, PGD inhibitor (PGDi) treatments ((-): DMSO, (+): 6AN) caused 6PG (right) but not G6P (left) to accumulate across the indicated PGD^{high} rapid autopsy subclones (n=3 technical replicates; error bars: s.d.m.). **b**, PGD inhibitor treatments recurrently increased MondoA nuclear:cytosolic ratios across the indicated PGD^{high} rapid autopsy subclones irrespective of the trafficking pattern (purely cytosolic to nuclear; purely cytosolic to diffuse; diffuse to nuclear). Green: MondoA IF signals, n=3 biological replicates; error bars: s.e.m.; indicated p-values calculated by two-tailed t-tests, scale bars: 20μm. **c**, Treatment of PGD^{high} cells with PGD inhibitor at the indicated time points (x-axis) caused 6PG (bottom) and TXNIP transcripts (middle) to accumulate with similar kinetics with corresponding slowing of glucose consumption rates (top) (n=3 technical replicates, error bars: s.d.m.).

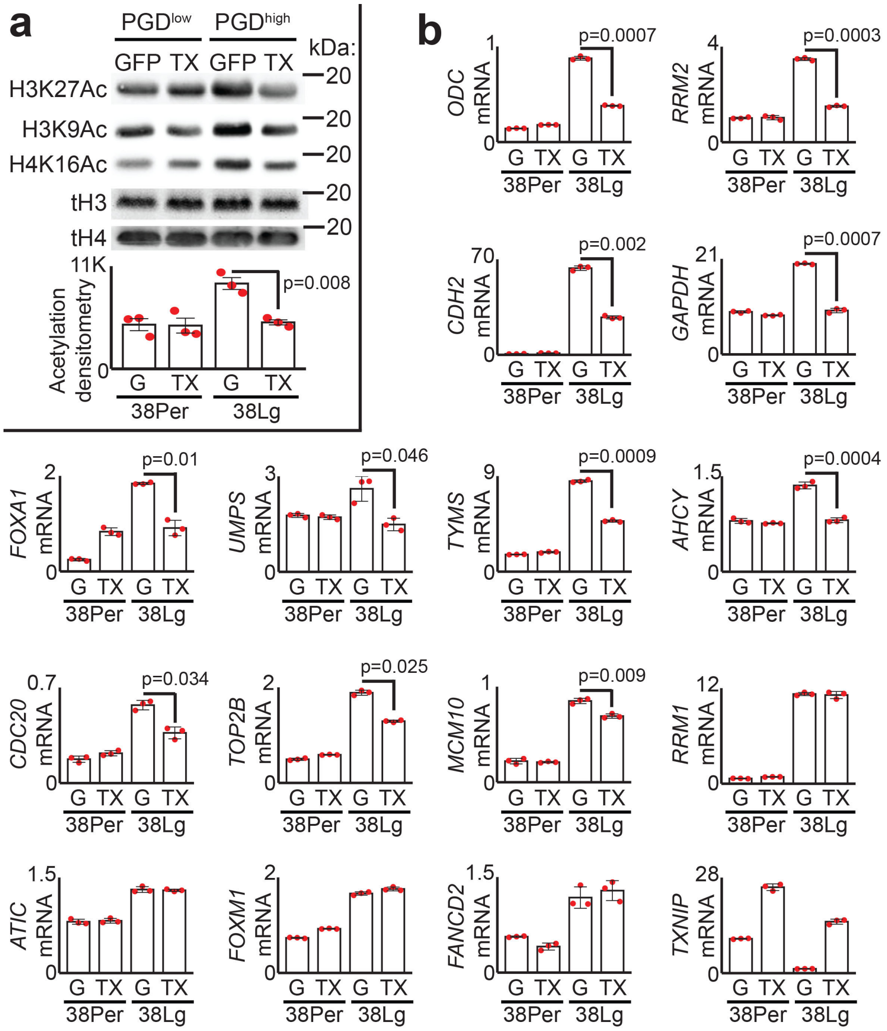

Supplementary Figure 9 Restoring TXNIP reverses histone hyperacetylation and represses metastatic transcripts. **a**, Western blots detected quantitative reductions of the indicated histone acetylation marks within bulk histones extracted from PGD^{high} cells expressing exogenous TXNIP (TX), relative to GFP-expressing controls and matched PGD^{low} cells (as quantified by densitometry, n=3 biological replicates, error bars: s.e.m., p=0.008 by two-tailed t-tests). **b**, RT-qPCR plots of the indicated gene transcript expression from PGD^{low} (38Per) and matched PGD^{high} (38Lg) cells expressing either exogenous GFP (G) or exogenous TXNIP (TX). This data is also summarized by the normalized Heatmap presented in Fig. 6c (n=3 technical replicates, error bars: s.d.m., indicated p-values calculated by two-tailed t-tests).

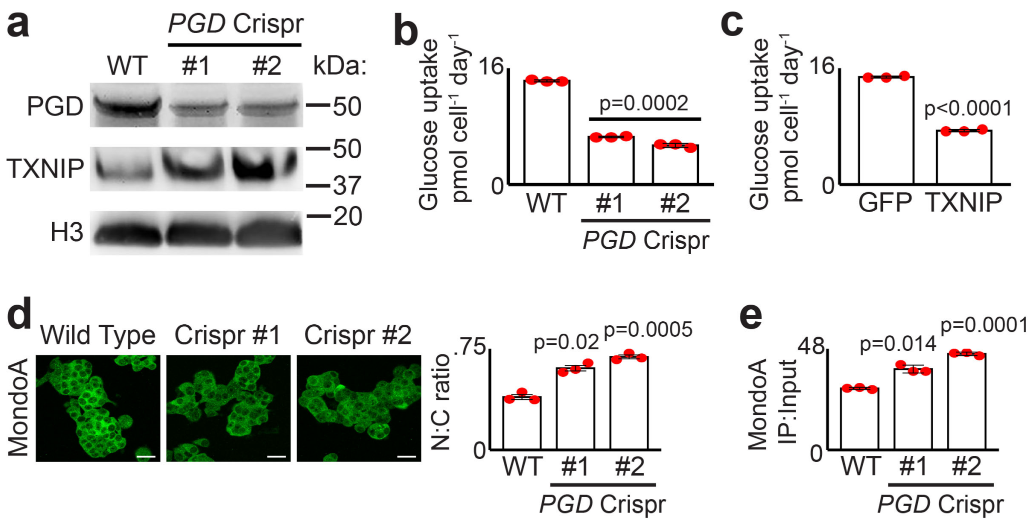

Supplementary Figure 10 Verification of PGD-dependent properties in 13Pr. a, Western blots confirmed that PGD Crispr sgRNAs (#1, #2, ref.[5]) lowered PGD protein expression and raised TXNIP expression compared to wild type (WT) 13Pr control cells. Blots are representative of two biological replicates. **b-c**, PGD inactivation by Crispr/Cas (panel b) or exogenous TXNIP expression (panel c) each slowed glucose consumption rates compared to wild type (WT) and GFP 13Pr control cells (n=3 technical replicates, error bars: s.d.m., p=0.0002 and p<0.0001 by two-tailed t-tests as indicated). **d,** PGD inactivation by Crispr/Cas increased MondoA nuclear:cytosolic ratios as expected (high cytosolic to diffuse trafficking pattern) by confocal IF (n=3 biological replicates, error bars: s.e.m., p=0.02 and 0.0005 by two-tailed t-tests as indicated). **e,** ChIP assays confirmed that MondoA was enriched at the TXNIP promoter during Crispr/Cas PGD inactivation (n=3 technical replicates, error bars: s.d.m., p=0.014 and 0.0001 by two-tailed t-tests as indicated).

Supplementary Table 1. Real-time PCR primer sequences and antibodies. ChIP annealing: 65°C, RT-qPCR annealing: 55°C.

ChIP Primers	Primer Location: Primer Sequence (5'-3')
CDH2 ChIP	Chr18_24.253F: GCTCAGCCCTGTATCAGCCAGC
CDH2 ChIP	Chr18_24.253R: GGGTTACAGGTATGAGCCACTGC
CDH2 ChIP	Chr18_24.506F: AATGGAGAAGTCAGGAATGTAGTCC
CDH2 ChIP	Chr18_24.506R: GTATTTCATTTATCAAGTTGCAGCTCC
CDH2 ChIP	Chr18_24.853F: TTTGCTTCTCACTCCAAGTTCATCC
CDH2 ChIP	Chr18_24.853R: CAACCTCAGGAACAATGCATCAGC
CDH2 ChIP	Chr18_25.125F: CGAAACAGTCCAGCTGCTATGG
CDH2 ChIP	Chr18_25.125R: CTTGGCTATTGTGACTGGTACTGC
CDH2 ChIP	Chr18_25.432F: CCAATGCACTAATTTAATGTCATGC
CDH2 ChIP	Chr18_25.432R: CGTGCTAATTTCTATGGTACACTGG
CDH2 ChIP	Chr18_25.632F: CCTAATCCAATATGCCTGGTGTCC
CDH2 ChIP	Chr18_25.632R: CTGGAAGTCTGAGATCAAGGTGC
CDH2 ChIP	Chr18_25.778F: AATAATCACGAAGCACTTCTGTATTGC
CDH2 ChIP	Chr18_25.778R: TCACCAGCAGACATAGTCATACTTCC
CDH2 ChIP	Chr18_25.808F: CCTTGGAGGTGGAGTCTACAGAGG
CDH2 ChIP	Chr18_25.808R: CTGCTAGCGTAGCCATCTGAGATCG
FOXA1 ChIP	Chr14_38.051F: AATATGCAAAGGAAGCGCAGCAGTCC
FOXA1 ChIP	Chr14_38.051R: GATGAGTTTTAAACTCTGGATTCTACAGG
FOXA1 ChIP	Chr14_38.052F: GGAGGAGGCGACATTCGTGTCAGG
FOXA1 ChIP	Chr14_38.052R: TCTCGCTTCCACGCCAGCCATTGC
FOXA1 ChIP	Chr14_38.055F: CAACCTCACACTTAAATTCTGAGTCAGG
FOXA1 ChIP	Chr14_38.055R: AAGGGGGAAATCCTCACTTGCCACC
FOXA1 ChIP	Chr14_38.058F: ATTTCTGGATTTTTTCAAGTGCTACATGG
FOXA1 ChIP	Chr14_38.058R: TCACCAGGGCTTGAACTTGGACAGC
FOXA1 ChIP	Chr14_38.060F: TTGCCACAGACCTGTAAACTCGTAGG
FOXA1 ChIP	Chr14_38.060R: CAATTTTTTTCCTGCGAAGTTTAATGATCC
FOXA1 ChIP	Chr14_38.060F: TTCAGGTGCAGCTGGGACTCGTGG
FOXA1 ChIP	Chr14_38.060R: CGCCTCGGAGTTGAAGACTCCAGC
FOXA1 ChIP	Chr14_38.062F: GATAGAGCAAATCACCTATGGACATGG
FOXA1 ChIP	Chr14_38.062R: CTTTTAACATTACAGTTGCTACAAGCAGG
FOXA1 ChIP	Chr14_38.067F: GCGATACTGGATTGAACTCCGCACG
FOXA1 ChIP	Chr14_38.067R: CAGGCTGCAGCCGCTGGACCTGG
ODC1 ChIP	Chr02_10.562F: TAGAACTGTTCCAAGCAGTGAGGAGC
ODC1 ChIP	Chr02_10.562R: GGGACTGGTGAGTGGCCACTGAGC
ODC1 ChIP	Chr02_10.564F: GTGCAGCCCTCGGGTGAGTTAAGC
ODC1 ChIP	Chr02_10.564R: GCAGATTCGGGCAGCGAGTGACC
ODC1 ChIP	Chr02_10.569F: GTGCCCTGGGCAGATCTAGCTTGC
ODC1 ChIP	Chr02_10.569R: CTGGAGAACCCTGACTAATGTAGTGG
ODC1 ChIP	Chr02_10.570F: GGTGTAGTTCCAGTCTGAATCTTAAGG

ODC1 ChIP	Chr02_10.570R: TACCTCCTCCATATCTGAGCAGAGG
ODC1 ChIP	Chr02_10.572F: CGTGCCAGGTCACGCCCTAGTGG
ODC1 ChIP	Chr02_10572R: GAAGGCCACGCCACGGATGAACC
ODC1 ChIP	Chr02_10.575F: TGAGTCGGGAGCTCTGGATCGAGG
ODC1 ChIP	Chr02_10.575R: GTGCCCTGTATTGTATCTGGCATTCC
ODC1 ChIP	Chr02_10.578F: CACAGCCCCGGCTACCGTCTGG
ODC1 ChIP	Chr02_10.579R: GAAACAATGCTCTTTCAGCCAGTTAGG
ODC1 ChIP	Chr02_10.584F: GAGTCCCAGGCTTCATGACTCAATGG
ODC1 ChIP	Chr02_10.584R: TTTATTCCACGTGTTAAGCCACATATGG
ODC1 ChIP	Chr02_10.594F: ACCCAACAAGTTGCAGCACCTCTGC
ODC1 ChIP	Chr02_10.594R: GGCACAGAGCAAGTGCTCACAGC
ODC1 ChIP	Chr02_10.597F: TGTTTCTTGCAAATGTCATGAGAGTTGG
ODC1 ChIP	Ch02_10.598R: AGAGAACTCCTGGTTCCACAGCTGG
TXNIP ChIP	ChoRE_F: CCAGGAGCACACCGTGTC
TXNIP ChIP	ChoRE_R: AGTTTCAAGCAGGAGGCG
**qRT-PCR Primers**	**Primer Sequence (5'-3')**
CDH2 F	TTATTACTCCTGGTGCGAGT
CDH2 R	GAGCTGATGACAAATAGCGG
TOP2B F	GTTACAGGTGGTCGTAATGGTT
TOP2B R	TTGGCTTCAGAAGTCTTCATCA
ODC1 F	TCTGATGACGAAGATGAGTC
ODC1 R	GGTTTAGGTCTCTTTTGCAG
RRM2 F	AGAGATGAGGGTTTACACTG
RRM2 R	ATTAGAGTGCAATTCATCCC
GAPDH F	ACAGTTGCCATGTAGACC
GAPDH R	TTTTTGGTTGAGCACAGG
UMPS F	TGATAACTCTGGCAAAATGC
UMPS R	AAGATACCTCCTTCATACTGC
TYMS F	GTTCTATGTGGTGAACAGTG
TYMS R	GGTAAATATGTGCATCTCCC
AHCY F	TGATGAGTAACTCCTTCACC
AHCY R	TTGGTCAACTTCACATTCAG
CDC20 F	CAGCTATATCCTGTCCAGTG
CDC20 R	CCAAGTTATCATTACCACCAC
MCM10 F	CTTATACAGAAGAGGCTGATG
MCM10 R	CCTCTTGCAACTCTTCATTC
SCD F	CAGAGGAGGTACTACAAACC
SCD R	ATAAGGACGATATCCGAAGAG
RRM1 F	GCTGTTTGTAATTTGGCTTC
RRM1 R	CCTCTGGTACAGGATAGTAG
ATIC F	CTCTATCCCTTTGTAAAGACAG
ATIC R	TCTCAGTAAGGTTACTCCAC
FOXM1 F	CATTGGACCAGGTGTTTTAAG

FOXM1 R	GGATAGGTACCAGGTATGAG
FOXA1 F	GATGGAAGGGCATGAAAC
FOXA1 R	GGCATAGGACATGTTGAAG
CDH1 F	TACATCTCCCTTCACAGC
CDH1 R	ATAGATTCTTGGGTTGGGTC
FANCD2 F	ACTCAGTGACCTACTGATAG
FANCD2 R	CTTCAATAGGAAGTTTGGGTC
SAT1 F	TCTGAAGAATCTAAGCCAGG
SAT1 R	CTCCTCTGTTGCCATTTTTAG
ATP1B1 F	CCAGGATTAACACAGATTCC
ATP1B1 R	GTTCAGTACATATGCCTCATAG
HMGCR F	TTATGTCTCTAGGCTTGGTTC
HMGCR R	CTTGGTTCAATTCTCTTGGAC
IDH1 F	TAAAGGTTTACCCAATGTGC
IDH1 R	CTGTAGACCTAGTTACCAAAAG
FASN F	CAATACAGATGGCTTCAAGG
FASN R	GATGTATTCAAATGACTCAGGG
ACSS2 F	TCAATGTATCTGGACACCTG
ACSS2 R	CTCTAATCTGCTTCTTGAGC
TXNIP F	CTGATCTATGTTAGCGTTCC
TXNIP R	TATCAGGGATGTTCAGATCTAC
PGD F	CATACCACCTGATGAAAGAC
PGD R	GCTCTGTCTTATTCCAATCC
**Antibodies**	**Source, Dilution**
TXNIP	Cell Signaling (D5F3E), 1:100 (IF); 1:250 (IHC)
MondoA	Proteintech (13614-1-AP), 1:100 (IF)
GLUT1	Millipore (07-1401), 1:100 (IF)
ACLY	Proteintech (15421-1-AP) 1:25 (IF)
H3K27Ac	Abcam (ab4729), 1:1000 (IF)
H4K16Ac	Abcam (ab109463), 1:1000 (IF)
