# Supplementary material for: Pancreatic cancers suppress negative feedback of glucose transport to reprogram chromatin for metastasis
Source: Nat Commun. 2020 Aug 13;11:4055. doi: 10.1038/s41467-020-17839-5 (PMC7426874; doi:10.1038/s41467-020-17839-5)
Supplement: Supplementary file 3 — Reporting Summary [file 41467_2020_17839_MOESM3_ESM.pdf]

nature research

Corresponding author(s): Oliver G McDonald

Last updated by author(s): 2020/07/07

Reporting Summary

Nature Research wishes to improve the reproducibility of the work that we publish. This form provides structure for consistency and transparency in reporting. For further information on Nature Research policies, see Authors & Referees and the Editorial Policy Checklist .

Statistics

For all statistical analyses, confirm that the following items are present in the figure legend, table legend, main text, or Methods section.

n/a	Confirmed	
☐	☒	The exact sample size (n) for each experimental group/condition, given as a discrete number and unit of measurement
☐	☒	A statement on whether measurements were taken from distinct samples or whether the same sample was measured repeatedly
☐	☒	The statistical test(s) used AND whether they are one- or two-sided<br>*Only common tests should be described solely by name; describe more complex techniques in the Methods section.*
☒	☐	A description of all covariates tested
☐	☒	A description of any assumptions or corrections, such as tests of normality and adjustment for multiple comparisons
☐	☒	A full description of the statistical parameters including central tendency (e.g. means) or other basic estimates (e.g. regression coefficient) AND variation (e.g. standard deviation) or associated estimates of uncertainty (e.g. confidence intervals)
☐	☒	For null hypothesis testing, the test statistic (e.g. F, t, r) with confidence intervals, effect sizes, degrees of freedom and P value noted<br>*Give P values as exact values whenever suitable.*
☒	☐	For Bayesian analysis, information on the choice of priors and Markov chain Monte Carlo settings
☒	☐	For hierarchical and complex designs, identification of the appropriate level for tests and full reporting of outcomes
☐	☒	Estimates of effect sizes (e.g. Cohen's d, Pearson's r), indicating how they were calculated

Our web collection on statistics for biologists contains articles on many of the points above.

Software and code

Policy information about availability of computer code

Data collection	Immunofluorescence imaging: Zeiss Zen v2.3 SP1 ; Histopathology imaging: Olympus cellSens Standard
Data analysis	Immunofluorescence quantification: ImageJ v1.52p ; Data plotting and statistical analyses: Microsoft Excel, Graphpad Prism v8.2.1, R v4.0

For manuscripts utilizing custom algorithms or software that are central to the research but not yet described in published literature, software must be made available to editors/reviewers. We strongly encourage code deposition in a community repository (e.g. GitHub). See the Nature Research guidelines for submitting code & software for further information.

Data

Policy information about availability of data

All manuscripts must include a data availability statement. This statement should provide the following information, where applicable:

- Accession codes, unique identifiers, or web links for publicly available datasets
- A list of figures that have associated raw data
- A description of any restrictions on data availability

Source data used to generate main and supplementary figure plots are provided in the Source Data File.
RNA-seq data were analyzed from previously published datasets (Hayashi et al. Nature Cancer 1(1):59–74, 2020). Those datasets are available through the European Genome Phenome Archive (EGA) at https://www.ebi.ac.uk/ega/studies/EGAS00001003974
SAGE datasets were analyzed from previously published data (Table S9B from Jones et al. Science 321(5897):1801-6, 2008). Those datasets are available at https://science.sciencemag.org/content/suppl/2008/09/04/1164368.DC1?_ga=2.119388298.2034492498.1593464458-1581683601.1591377453

Field-specific reporting

Please select the one below that is the best fit for your research. If you are not sure, read the appropriate sections before making your selection.

- [x] Life sciences
- [ ] Behavioural & social sciences
- [ ] Ecological, evolutionary & environmental sciences

For a reference copy of the document with all sections, see nature.com/documents/nr-reporting-summary-flat.pdf

Life sciences study design

All studies must disclose on these points even when the disclosure is negative.

Sample size	Sample sizes were determined by the number of samples available for analysis
Data exclusions	RNA-seq analyses (from Hayashi et al. Nature Cancer 2020) were conducted to compare TXNIP expression in matched primary tumor regions and liver metastases from the same individual patient(s). Patients that did not have matched primary tumor and liver metastasis data available were therefore excluded from the analysis.
Replication	Experiments were reproduced at least 3 times with statistical tests of significance. All data could be reproduced.
Randomization	The study was not an experimental or clinical trial, and therefore no randomization was performed. The cell line and patient tissue samples were not subdivided prior to analysis. IHC studies were performed on retrospectively collected specimens.
Blinding	Immunohistochemical stained sections were scored by a pathologist who was blinded to the source of the specimen (liver, lung, peritoneum).

Reporting for specific materials, systems and methods

We require information from authors about some types of materials, experimental systems and methods used in many studies. Here, indicate whether each material, system or method listed is relevant to your study. If you are not sure if a list item applies to your research, read the appropriate section before selecting a response.

Materials & experimental systems

n/a	Involved in the study	
[ ]	[x]	Antibodies
[ ]	[x]	Eukaryotic cell lines
[x]	[ ]	Palaeontology
[ ]	[x]	Animals and other organisms
[ ]	[x]	Human research participants
[x]	[ ]	Clinical data

Methods

n/a	Involved in the study	
[x]	[ ]	ChIP-seq
[x]	[ ]	Flow cytometry
[x]	[ ]	MRI-based neuroimaging

Antibodies

Antibodies used	Rabbit monoclonal TXNIP (Cell Signaling D5F3E)<br>Rabbit polyclonal MondoA (Proteintech 13614-1-AP)<br>Rabbit polyclonal GLUT1 (Millipore 07-1401)<br>Rabbit polyclonal ACLY (Proteintech 15421-1-AP)<br>Rabbit polyclonal H3K27Ac (Abcam ab4729)<br>Rabbit monoclonal H4K16Ac (Abcam ab109463)
Validation	IHC experiments using TXNIP antibodies on formalin fixed paraffin embedded patient tissues were performed and validated in the Vanderbilt Translational Pathology Shared Resource (TPSR) using positive and negative control tissues and isotype controls with appropriate staining. Validation statements of commercially available antibodies are available at the manufacturer's websites: MondoA: https://www.ptglab.com/products/MLXIP-Antibody-13614-1-AP.htm#validation ; TXNIP: https://www.cellsignal.com/products/primary-antibodies/txnip-d5f3e-rabbit-mab/14715 ; GLUT1: https://www.emdmillipore.com/US/en/product/Anti-GLUT-1-Antibody-CT,MM_NF-07-1401 ; ACLY: https://www.ptglab.com/products/ACLY-Antibody-15421-1-AP.htm#validation ; H3K27Ac: https://www.abcam.com/histone-h3-acetyl-k27-antibody-chip-grade-ab4729.html ; H4K16Ac: https://www.abcam.com/histone-h4-acetyl-k16-antibody-epr1004-ab109463.html

Eukaryotic cell lines

Policy information about cell lines

Cell line source(s)	2Lv, 2Lg, 6Lv, 10Lv, 13Pr, 13Lg, 32O, 38Lv, 38Lg, 38Per, 125Lv are primary cell lines previously isolated from patients through the Johns Hopkins Rapid Autopsy Medical Donation Program (Embuscado et al. Cancer Biology & Therapy 4(5):548-554, 2005 ; Yachida et al. Nature 467:1114–1117, 2010 ; McDonald et al. Nature Genetics 49(3):367-376, 2017) ; HPDE, HPAF-II, ASPC-1, 2.13, 3.27, 5.04 cell lines were purchased commercially from ATCC.
Authentication	Sequencing, STR, experimental monitoring of characteristics known to be unique to each line
Mycoplasma contamination	All cell lines negative for mycoplasma (Sigma Lookout)
Commonly misidentified lines (See ICLAC register)	No commonly misidentified cell lines were used in this study

Animals and other organisms

Policy information about studies involving animals; ARRIVE guidelines recommended for reporting animal research

Laboratory animals	NU/J (nude atymic), equal male and female mice, aged 6-10 weeks. All mice were housed under sterile conditions, 21.9 +/- 0.8 degrees celsius, 45+/=15% humidity, and 12 hour light/dark cycle (7am-7pm light; 7pm-7am dark).
Wild animals	No wild animals were used for this study.
Field-collected samples	No Field-collected samples were used for this study.
Ethics oversight	Animal study protocols were advised and approved by the Institutional Animal Care and Use Committee (IACUC) within the Division of Animal Care (DAC) at Vanderbilt University Medical Center.

Note that full information on the approval of the study protocol must also be provided in the manuscript.

Human research participants

Policy information about studies involving human research participants

Population characteristics	Vanderbilt patient samples used for IHC studies: 55 total patients (34 with liver or lung metastasis; 21 with peritoneal metastasis), 30/55 male, 39/55 Caucasian, 4/55 treated with chemotherapy, median age 63 (range: 35-83). Detailed demographic information of the additional previously published rapid autopsy patients included in this study including gender, race, age, and therapy status are available in Table S1 from Hayashi et al. Nature Cancer 1(1):59–74, 2020.
Recruitment	For IHC studies, formalin fixed paraffin embedded tissue samples from patients with metastatic pancreatic cancer were retrospectively retrieved from the pathology archives at Vanderbilt University Medical Center from the years 2013-2018. Rapid Autopsy cell lines (Johns Hopkins) and frozen tissues (Memorial Sloan Kettering) were collected from organs prospectively donated by patients to the Rapid Autopsy Medical Donation Programs at those institutions.
Ethics oversight	Slides and formalin fixed paraffin embedded tissue blocks for IHC were collected with approval from the Vanderbilt Institutional Review Board. Written consent was not required for retrospective retrieval of tissue blocks. Cell lines and rapid autopsy tissues were collected from rapid autopsies with approval of the Johns Hopkins Institutional Review Board and Memorial Sloan Kettering Cancer Review Board after informed and written consent were obtained, following all relevant ethical regulations.

Note that full information on the approval of the study protocol must also be provided in the manuscript.
